# Angelicae Gigantis Radix Regulates LPS-Induced Neuroinflammation in BV2 Microglia by Inhibiting NF-κB and MAPK Activity and Inducing Nrf-2 Activity

**DOI:** 10.3390/molecules24203755

**Published:** 2019-10-18

**Authors:** You-Chang Oh, Yun Hee Jeong, Wei Li, Younghoon Go

**Affiliations:** Korean Medicine (KM)-Application Center, Korea Institute of Oriental Medicine, Daegu 41062, Korea; ulivuli@kiom.re.kr (Y.-C.O.); runxi0333@kiom.re.kr (Y.H.J.); liwei1986@kiom.re.kr (W.L.)

**Keywords:** Angelicae Gigantis Radix, antineuroinflammation, Nrf-2, NF-κB, MAPK

## Abstract

Angelicae Gigantis Radix (AGR) has been widely used as a traditional medicine in East Asia. The effects of AGR on neuroinflammation have not previously been studied in detail. In the study presented here, we investigated the antineuroinflammatory properties of this herb and its mechanism of operation. The effects of AGR on neuroinflammation were studied by measuring the production of inflammatory factors and related enzymes, and analyzing the expression levels of proteins and genes involved its activity, in lipopolysaccharide (LPS)-stimulated BV2 microglia. We found that AGR pretreatment strongly inhibits the production of nitric oxide (NO), cytokines, and the enzymes inducible nitric oxide synthase (iNOS), and cyclooxygenase (COX)-2, and effectively induces the activation of heme oxygenase (HO)-1 and its regulator, nuclear factor erythroid 2-related factor 2 (Nrf-2). We also found that AGR effectively regulates the activation of nuclear factor (NF)-κB and mitogen-activated protein kinase (MAPK). We confirmed the antineuroinflammatory effects of the main constituents of the plant as identified by high-performance liquid chromatography (HPLC). Our results indicate that the neuroinflammation inhibitory activity of AGR occurs through inhibition of NF-κB and MAPK and activation of Nrf-2.

## 1. Introduction

Neuroinflammation is one part of the immune reaction to harmful stimulation in the central nervous system. The function of the neuroinflammatory response is to remove necrotic cells and tissues caused by pathogenic infections, disease, and other damage. Uncontrolled neuroinflammation, however, contributes to the progress of neurodegenerative diseases including Parkinson’s disease, Alzheimer’s disease, and Huntington’s disease [1]. Microglial cells, brain-specific macrophages that reside in the central nervous system (CNS), play a critical role in maintaining the homeostasis of the brain and the neuroinflammatory response [2]. Microglia contribute to the maintenance of synaptic homeostasis by removing debris such as damaged neurons and pathogens from the central nervous system [3].

Abnormal activation of microglia by inflammatory stimuli, such as LPS, leads to the secretion of neurotoxic molecules including NO, prostaglandin (PG) E_2_, and inflammatory cytokines. Overexpression of these inflammatory factors causes damage to nerve cells and ultimately leads to neurodegenerative disease [4]. Recently, a number of studies have aimed at identifying agents which control the activation of microglial cells, with the ultimate aim of preventing or treating brain diseases associated with chronic inflammation. Some of these studies have involved natural substances.

Microglial cells respond to LPS stimulation by secreting inflammatory factors such as NO, PGE_2_, and the proinflammatory cytokines interleukin (IL)-6, IL-1β, and tumor necrosis factor (TNF)-α [5]. Levels of NO and PGE_2_ are closely related to the expression of the enzymes which synthesize them, iNOS and COX-2. COX-2 and iNOS are important for the progression of several inflammatory diseases, in which iNOS production is suppressed by HO-1, an important component of the immune system, resulting in a negative feedback loop [6]. Increases in the amount of HO-1 in activated microglia reduce the levels of proinflammatory factors such as NO, TNF-α, IL-6, and IL-1β, and the production of HO-1 is regulated by a redox-dependent transcription factor, Nrf-2 [7]. In the activation phase of the inflammatory reaction, free Nrf-2 is translocated into the nucleus, where it binds to the antioxidant response element, which in turn induces the production of HO-1 [8]. Some recent studies show that HO-1 is important for immunomodulation and anti-inflammatory effects.

NF-κB is an important transcription factor involved in inflammatory processes and the regulation of inflammatory factors such as cytokines, chemokines, inducible enzymes, growth factors, and immune receptors [9]. NF-κB is potentially an important molecular target for the development of agents against inflammatory diseases, and many recent studies have focused on the regulation of NF-κB. The activity of NF-κB is closely related to that of the MAPK signaling pathway [10]. MAPK is an intracellular enzyme that activates inflammatory processes in response to stimuli such as LPS from the extracellular environment. MAPK includes an extracellular signal-regulated kinase (ERK), p38, and members of the c-Jun NH_2_-terminal kinase (JNK) subfamily. It plays a role in the regulation of inflammatory molecules and immune-related cytotoxic factors that induce expression of inflammatory gene in microglia or macrophages [11]. MAPK, along with NF-κB, is therefore an important potential target for the treatment of inflammatory diseases [12].

AGR is the root of *Angelica gigas* Nakai (Umbelliferae), known as “Danggui” in Korea. AGR is cultivated as a medicinal plant over a wide area of Asia, but is mainly produced in Korea, China, and Japan. Plants of different origin are used in the three countries. *Angelica gigas* Nakai is the “Danggui” commonly used in Korea. AGR is listed in the ancient medicine text “Shennong’s Classic of Materia Medica,” and is widely used in traditional Asian medicine for improving blood circulation and hematopoiesis. Recent research has shown that AGR promotes blood flow in the coronary arteries and stimulates red blood cell generation. However, the effect of AGR on neuroinflammation mediated by microglial cells and its effects on NF-κB, MAPK, and Nrf-2 has not previously been studied.

In this study, we investigated the effect of an ethanol extract of AGR on the inflammatory response using LPS stimulation in brain microglia BV2 cells. We also investigated how the potency of the extracts correlated with the activation or inactivation of the NF-κB, MAPK, and Nrf-2 signaling pathways. We investigated the chemical constituents of AGR ethanol extracts using HPLC.

## 2. Results

### 2.1. Effect of AGR Extract on the Viability of BV2 Microglial Cells

**Figure 1:**
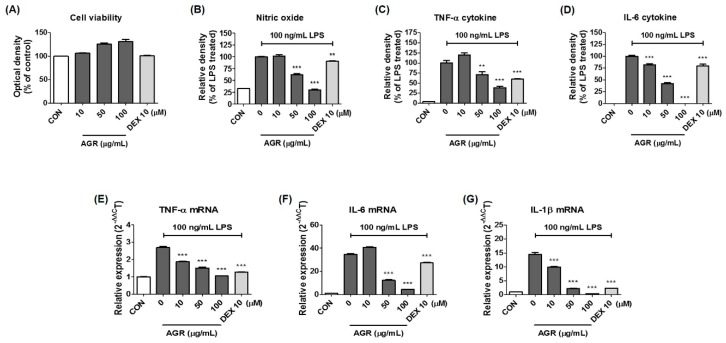
Effects of AGR on (**A**) cell viability, secretion of (**B**) NO, (**C**,**D**) inflammatory cytokines, and (**E**–**G**) mRNA expression in BV2 microglia. Control cells were incubated with vehicle alone. Data represent the mean ± SEM of determinations from three independent experiments. *p* values (** *p* < 0.001 and *** *p* < 0.0001) were calculated from comparisons with LPS stimulation values.

Cell viability tests using the cell counting kits (CCK) reagent showed no cytotoxicity to BV2 cells when treated with 10–100 μg/mL of ARG, and slight proliferation was observed at 50 and 100 μg/mL (Figure 1A). Subsequent experiments evaluating the effect of AGR on neuroinflammation induced by LPS in microglial cells used concentrations of 100 μg/mL or less, to avoid any potential cytotoxic effects while retaining some efficacy.

### 2.2. Inhibitory Effect of AGR on NO Secretion by Microglial Cells

Griess assays were performed to investigate the effects of AGR extract on NO secretion. NO is one of the final products of the neuroinflammatory reaction, and is synthesized from L-arginine through the catalytic activity of the enzyme iNOS. In this and subsequent experiments, the efficacy of AGR was compared to that of 10 μM dexamethasone (DEX), a steroidal anti-inflammatory drug, which was used as a positive control. BV2 cells showed an increase in NO secretion after LPS stimulation (reached at 41.68 ± 0.26 μM), and showed a pattern of inhibition which was dependent upon the pretreatment concentration of the AGR extract (Figure 1B). Statistical significance was evident at concentrations of 50 μg/mL or higher, and pretreatment with DEX produced only a slight inhibitory effect.

### 2.3. Inhibitory Effects of AGR on Levels of the Proinflammatory Cytokines TNF-α and IL-6

The effects of AGR on the secretion of proinflammatory cytokines was investigated, to determine the efficacy of AGR for the inhibition of neuroinflammation at the cellular level. Levels of the cytokines TNF-α and IL-6 in BV2 cells increased after LPS stimulation (TNF-α: 1522.90 ± 190.73 pg/mL and IL-6: 1814.98 ± 66.78 pg/mL), and cytokine secretion was decreased after pretreatment with AGR. TNF-α secretion was strongly inhibited at concentrations above 50 μg/mL (Figure 1C), and IL-6 was significantly dose-dependently inhibited at all concentrations (Figure 1D). In particular, the application of 100 μg/mL of AGR inhibited IL-6 secretion almost completely. DEX treatment inhibited TNF-α and IL-6 secretion at statistically significant levels.

### 2.4. Effect of AGR on Cytokine mRNA Expression

After establishing the inhibitory effect of AGR on the secretion of NO and inflammatory cytokines, we examined the effect of pretreatment with AGR extract on the expression of cytokine mRNA. As seen in Figure 1C,D, the expression of TNF-α and IL-6 mRNA was significantly inhibited by pretreatment with AGR, and a concentration-dependent pattern of expression was observed (Figure 1E,F). The expression of IL-1β mRNA was also suppressed (Figure 1G). The positive control DEX also produced significant inhibitory activity.

### 2.5. Inhibitory Effect of AGR Pretreatment on the Expression of iNOS and COX-2 Enzymes

**Figure 2:**
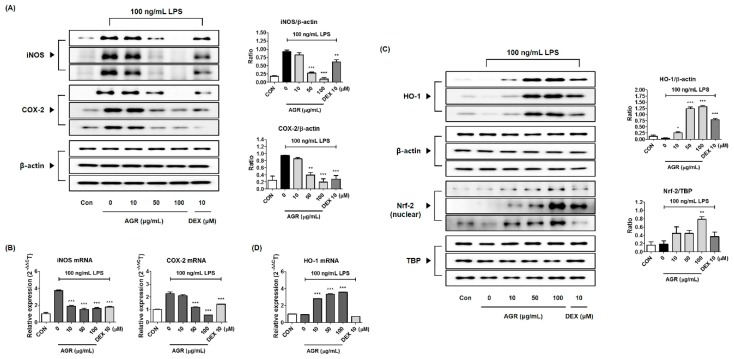
Effects of AGR on (**A**) protein production, (**B**) mRNA expression of iNOS, COX-2, and (**C**,**D**) production of HO-1, Nrf-2. Microglia cells were stimulated with LPS for (**A**,**B**) 12 h, (**C**) 6 h (HO-1) or 3 h (Nrf-2), or (**D**) 3 h. The histograms show protein and mRNA expression levels relative to those of β-actin or TBP. Data represent the mean ± SEM of three independent experiments. * *p* < 0.05, ** *p* < 0.001, and *** *p* < 0.0001 were calculated from comparisons with LPS stimulation values.

iNOS and COX-2 are enzymes which synthesize the inflammatory factors NO and PGE_2_ respectively, and are considered to be important in inflammatory responses. The expression of iNOS and COX-2 was significantly increased by the activation of inflammatory responses in BV2 microglial cells under LPS treatment, and pretreatment with AGR extract produced a concentration-dependent inhibition (Figure 2A). Real-time quantitative polymerase chain reaction (RT-qPCR) showed significant inhibition of iNOS and COX-2 mRNAs expression at concentrations of 50 μg/mL or more (Figure 2B).

### 2.6. Induction Efficacy of AGR on Anti-Inflammatory Factor HO-1 and its Molecular Regulator, Nrf-2

HO-1 induces biliverdin, bilirubin, and carbon monoxide (CO) that has antioxidant and anti-inflammatory effects by promoting oxidation of heme, and is also induced by pretreatment with AGR extracts even under conditions of LPS treatment. As shown in Figure 2C,D, HO-1 protein levels were increased after treatment with concentrations of AGR of 50 and 100 μg/mL (increase 10.30 and 11.03 times respectively compared to Con), and mRNA was expressed at treatment levels of 10, 50, and 100 μg/mL (increase 2.82, 3.35, and 3.59 times, respectively, compared to Con). Translocation of Nrf-2 into the nucleus, required for the regulation of the induction of HO-1, was also increased by pretreatment of AGR in a similar way to HO-1, and the amount of translocation gradually increased with concentration (Figure 2C). β-actin was used as the loading control for whole cell protein and TATA box-binding protein (TBP) was used for nuclear protein.

### 2.7. Effects of AGR Extract Pretreatment on NF-κB Pathway Activation

**Figure 3:**
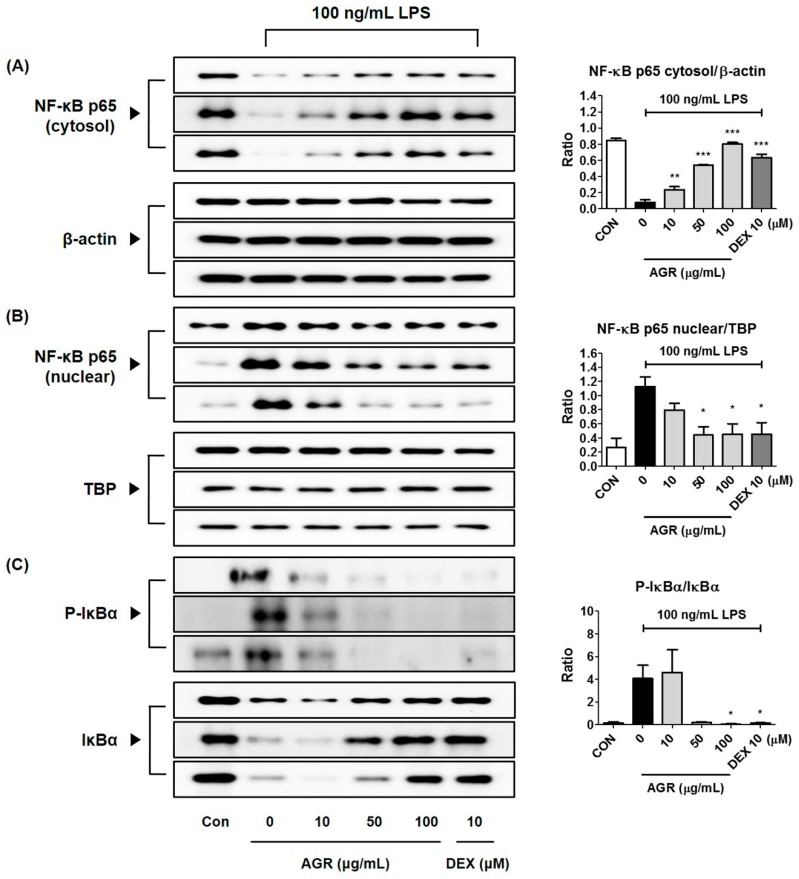
Effects of AGR on NF-κB p65 (**A**) cytosol, (**B**) nuclear protein expression, and (**C**) phosphorylation and degradation of IκBα. Cells were stimulated with LPS for (**A**,**B**) 1 h or (**C**) 30 min. The histograms show protein expression levels relative to those of β-actin or TBP. Data represent the mean ± SEM of three independent experiments. * *p* < 0.05, ** *p* < 0.001, and *** *p* < 0.0001 were calculated from comparisons with LPS stimulation values.

NF-κB, one of the transcription factors of the toll-like receptor (TLR) 4 signaling pathway, is strongly activated and translocated into the nucleus by treatment with LPS, and subsequently can promote the production of a variety of neuroinflammatory genes and factors, directly affecting the inflammatory response. To investigate how pretreatment with AGR extract regulates this activation, we examined cytoplasmic and nuclear expression levels of the p65 protein, and the changes in levels of inhibitor of NF-κB alpha (IκBα), an inhibitor protein of NF-κB. The amount of p65 protein in the cytoplasm decreased to basal levels after the initiation of the inflammatory response by LPS stimulation, but recovered after pretreatment with AGR extract (Figure 3A). The p65 protein level in the nucleus significantly increased after the LPS stimulation, and seems to be decreased by treatment with AGR extract, showing an effect pattern opposite to cytosolic p65 expression (Figure 3B). Based on these results, we confirmed that AGR effectively regulates NF-κB activity. Each protein sample underwent normalization using β-actin and TBP. The expression pattern of IκBα, an inhibitory protein, was also analyzed in order to confirm its regulatory effect on NF-κB nuclear transfer. IκBα, which is bound to NF-κB in the cytoplasm of normal cells, was degraded and strongly phosphorylated by LPS, and was inhibited by AGR and DEX, confirming the regulatory activity of AGR on NF-κB (Figure 3C).

### 2.8. Inhibitory Effect of AGR Extract on MAPK Phosphorylation in Microglial Cells Induced by LPS Stimulation

**Figure 4:**
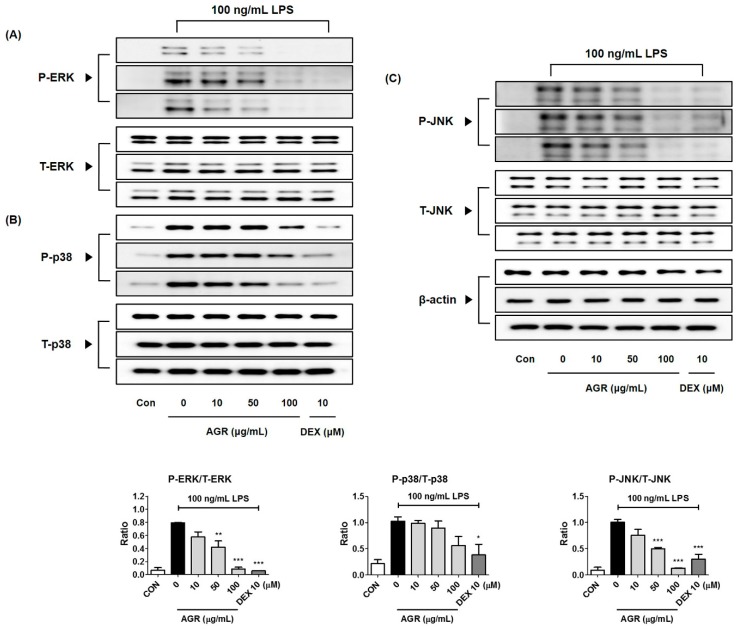
Effects of AGR on phosphorylation of (**A**) ERK, (**B**) p38, and (**C**) JNK MAPK. Cells were stimulated with LPS for 30 min. The histograms show protein expression levels relative to those of total-type protein. Data represent the mean ± SEM of three independent experiments. * *p* < 0.05, ** *p* < 0.001, and *** *p* < 0.0001 were calculated from comparisons with LPS stimulation values.

MAPK has four subfamilies, ERK1/2, p38, JNK, and ERK5, and is known to be an important part of the TLR4 channel, in conjunction with NF-κB, and is a major target for the development of medicine for many inflammatory diseases. In this study, we confirmed that activation of the inflammatory response of BV2 brain microglia by stimulation with LPS results in phosphorylation of three MAPKs, including ERK1/2, p38, and JNK. We tested the effect of three concentrations of AGR, 10, 50, and 100 μg/mL, on the phosphorylation state of each MAPK subfamilies. Phosphorylation of ERK was strongly inhibited by AGR, and was observed to have a highly concentration-dependent pattern (Figure 4A). Phosphorylation of p38 was inhibited only by treatment with the highest concentration, 100 μg/mL, but showed a suppression rate close to 50% (Figure 4B). JNK phosphorylation was inhibited by treatment with AGR in concentrations of more than 50 μg/mL in a concentration-dependent manner (Figure 4C). Pretreatment with AGR extract effectively regulated the phosphorylation of all three MAPK proteins in a concentration-dependent manner. The controls for ERK, p38, and JNK MAPK phosphoprotein used each total protein.

### 2.9. Identification of the Components of AGR using HPLC-Diode Array UV/VIS Detector (DAD) Analysis

**Figure 5:**
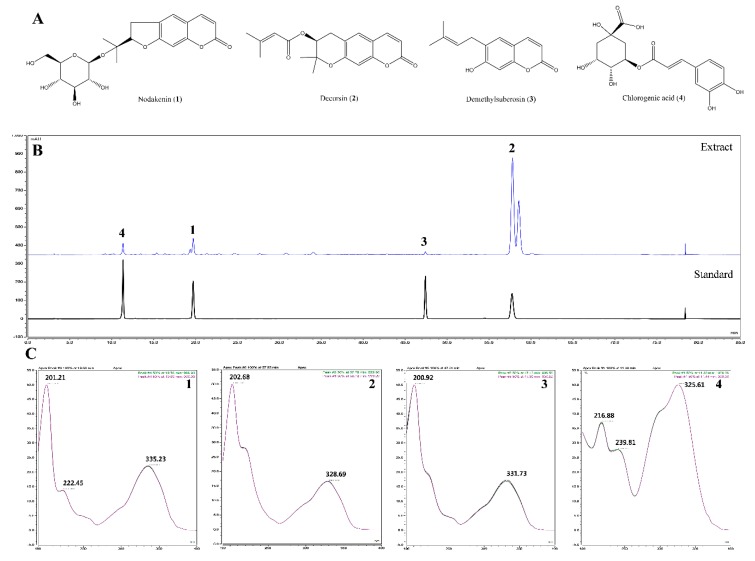
Structures of the four major compounds (**A**), HPLC chromatograms (**B**) of four standard compounds from the EtOH extract of *A. gigas* identified at UV wavelengths of 330 nm (**C**). nodakenin (1), decursin (2), demethylsuberosin (3), and chlorogenic acid (4) were identified.

In the study described here, the mobile phase consisted of acetonitrile (ACN) and water, and a C18 column was used as the stationary phase. Analytes were detected at wavelengths of 200–400 nm using a PDA detector to examine multiple classes of compounds. A wavelength of 330 nm was selected for detection because it produced better baseline and higher resolution in the chromatograms than in other wavelengths. As shown in Figure 5, the standards had retention times which included nodakenin (**1**; 19.71 min), decursin (**2**; 57.74 min), demethylsuberosin (**3**; 47.41 min), and chlorogenic acid (**4**; 11.36 min). All standards showed good selectivity without interference within 70 min. The identification of four compounds of AGR was based on comparison of chromatograms with each standard, retention times (tR), and UV spectra.

### 2.10. Validation of the Analytical HPLC Method

By comparing the chromatographic profile of the standards, the analytical HPLC method of the sample extract was selected. The calibration curves for the four major compounds were obtained via plotting the peak area versus the concentration using least-squares regression analysis. Each calibration equation was acquired using five concentrations ranging from 12.5 to 200 μg/mL for nodakenin (**1**) and chlorogenic acid (**4**), 37.5 to 600 μg/mL for decursin (**2**), and 3.125 to 50 μg/mL for demethylsuberosin (**3**). That means the range of all calibration curves (**1**–**4**) was adequate for the simultaneous determination of constituents in the sample extract. The linear correlation coefficient (*R*^2^) for the calibration curves was greater than 0.99, indicating a strong linear relationship (Table 1). According to the calibration curves, the amounts of compounds **1**–**4** in AGR were found to be 9.0498 ± 0.0241, 77.4079 ± 0.1176, 1.5062 ± 0.00013, and 5.3723 ± 0.00042 mg/g, respectively. These results indicate that this method of validation may be applied to identify traces of compounds in the extract.

### 2.11. Verification of Neuroinflammatory Inhibitory Efficacy and its Mechanism of the Three Main Constituents of AGR in BV2 Cells Stimulated by LPS

**Figure 6:**
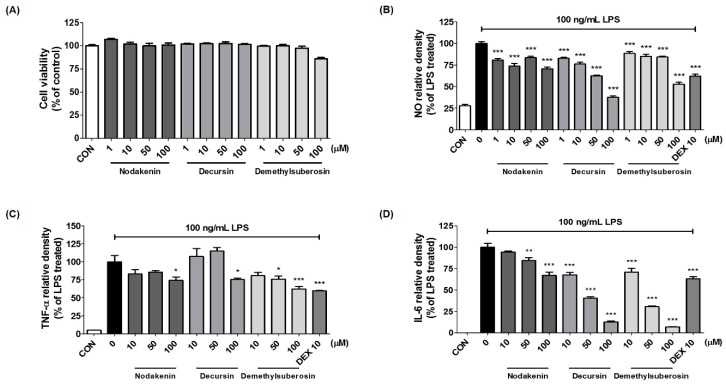
Effects of three compounds nodakenin, decursin, and demethylsuberosin on (**A**) cell viability and the secretion of (**B**) NO, (**C**) TNF-α, and (**D**) IL-6 cytokines. Data represent the mean ± SEM of duplicate determinations from three independent experiments. * *p* < 0.05, ** *p* < 0.001, and *** *p* < 0.0001 were calculated from comparisons with LPS stimulation values.

**Figure 7:**
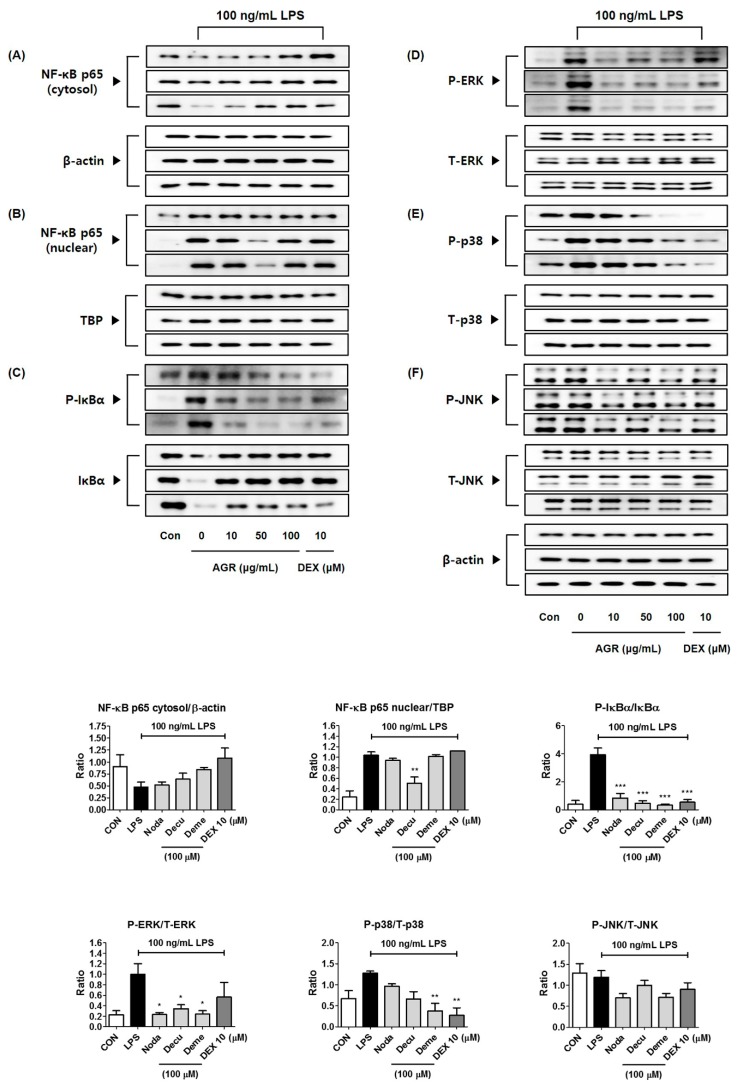
Effects of three compounds, nodakenin, decursin, and demethylsuberosin, on (**A**,**B**) nuclear translocation of NF-κB p65, (**C**) phosphorylation and degradation of IκBα, and phosphorylation of (**D**) ERK, (**E**) p38, and (**F**) JNK MAPK. Cells were stimulated with LPS for (**A**,**B**) 1 h or (**C**–**F**) 30 min. The histograms show protein expression levels relative to those of β-actin, TBP, or total-type protein. Data represent the mean ± SEM of three independent experiments. * *p* < 0.05, ** *p* < 0.001, and *** *p* < 0.0001 were calculated from comparisons with LPS stimulation values.

We used microglial cells stimulated by LPS to confirm the neuroinflammatory inhibitory activity of the three main components of AGR extracts identified through HPLC analysis: nodakenin (**1**), decursin (**2**), and demethylsuberosin (**3**). We measured the influence on viability of BV2 cells using CCK assays. Nodakenin and decursin did not show cytotoxicity at any of the concentrations used, from 1–100 μM, while demethylsuberosin showed weak cell toxicity only at 100 μM. At this concentration, survival was 85% or higher (Figure 6A). The effects of the three components on NO, and TNF-α, IL-6 cytokine secretion were measured using Griess assays and enzyme-linked immunosorbent assay (ELISA). Nodakenin and demethylsuberosin showed weak inhibitory effects on NO secretion, while decursin had a stronger, concentration-dependent inhibitory activity (Figure 6B). The three components nodakenin, decursin, and demethylsuberosin showed weak effects by inhibiting the secretion of TNF-α cytokine at the highest concentration (100 μM) by 26%, 25%, and 38%, respectively, and they showed statistical significance only at the highest concentration (Figure 6C). The secretion of IL-6 was effectively inhibited by nodakenin, decursin, and demethylsuberosin concentration-dependently, with decursin and demethylsuberosin showing especially strong inhibitory rates of 87% and 93%, respectively, at 100 μM (Figure 6D). We further assessed the effects on NF-κB and MAPK activation to explore the mechanism of neuroinflammatory suppressive activity of the three components. The highest concentration of each compound, 100 μM, was used, with the result that decursin showed strong inhibitory efficacy against nuclear translocation of NF-κB p65 (Figure 7A,B). Phosphorylation and degradation of IκBα were significantly inhibited by all three compounds (Figure 7C). In addition, after testing the effects of nodakenin, decursin, and demethylsuberosin on phosphorylation of ERK, p38, and JNK MAPK, all three compounds effectively inhibited phosphorylation of ERK (Figure 7D). Phosphorylation of p38 was significantly inhibited by demethylsuberosin alone, and phosphorylation of JNK was weakly inhibited by all three components (Figure 7E,F).

## 3. Discussion

Microglia are phagocytic cells located in the CNS which regulate the initial immune response in the brain. They secrete NO, inflammatory cytokines, reactive oxygen species, and superoxide generated by uncontrolled excessive inflammatory reactions, and contribute to the development of degenerative brain diseases such as dementia [13]. Therefore, the modulation of inflammatory mediators produced from microglia activated by excessive inflammatory responses has been identified as a therapeutic target in conjunction with amyloid beta (Aβ) and tau protein in recent studies of dementia. LPS, which is an endotoxin produced by Gram-negative bacteria, is a major producer of inflammation and specifically activates TLR4 pathway in macrophages and microglia cells, leading to the overproduction of NO, PGE_2_, and proinflammatory cytokines, including IL-6, IL-1β, TNF-α, macrophage inflammatory protein (MIP)-1, monocyte chemoattractant protein (MCP)-1, and IL-16 [14,15]. These inflammatory mediators act as cytotoxic substances, which accelerate the inflammatory response and contribute to the initiation and maintenance of chronic inflammatory diseases [15]. Therefore, effective control of the LPS-induced secretion of various inflammatory mediators, activation of enzymes, expression of related genes, and activation of the TLR4 mechanism is very important for preventing and treating not only inflammatory diseases of the CNS but also degenerative brain diseases. In this study, we investigated the effect of AGR, which has been widely used in food and medicine from ancient times in East Asia, on the inflammatory responses induced by LPS in BV2 microglia, in order to confirm the antineuroinflammatory activity of AGR and to analyze its mechanism.

Cultured BV2 cells were treated with AGR and incubated for 24 h to identify the cytotoxicity-free range of 100 μg/mL or less (Figure 1A). The inhibitory effect of AGR on NO secretion was then tested at lower concentrations. NO is an active nitrogen species that is a toxic low-molecular radical involved in the defense functions of the immune system [16]. NO is produced from L-arginine through constitutive nitric oxide synthase (cNOS) and iNOS. Excessive secretion of NO in the body causes cytotoxicity and mediates inflammatory responses in the CNS, leading to the onset of degenerative brain diseases [17]. Proinflammatory cytokines play a diverse role in immune, infectious, and tissue repair processes, cell growth, and biological defenses, and are involved in the pathogenesis of many diseases that are directly or indirectly related to inflammation. BV2 microglia activated by LPS secrete cytokines including IL-6, IL-1β, TNF-α, and transforming growth factor (TGF)-β, which may act to promote inflammation. TNF-α and IL-1β are typical proinflammatory cytokines associated with degenerative diseases, and they induce inflammatory disease by affecting the synthesis of mediators such as PG, leukotriene, and NO [18]. The levels of these cytokines are increased in the blood and brain tissue of patients with degenerative brain diseases, and they are known to play a pivotal role in the pathogenesis of neuronal apoptosis [19]. According to the results of this study, pretreatment with AGR extract significantly decreased the amount of NO secreted from BV2 cells after LPS stimulation (Figure 1B), due to the inhibition of mRNA and protein production of the iNOS protein (Figure 2A,B). Some members of the PG family are known to exacerbate progressive inflammation and pain [12]. PGE_2_ and PGD_2_, members of this family, are synthesized from arachidonic acid via the action of COX and lipoxygenase [14]. COX-2, one of the COX isomers, is a major target of research into therapeutic agents such as nonsteroidal anti-inflammatory drugs, and considerable research has been conducted into COX-2-selective inhibitors. In this study, BV2 cells were shown to express COX-2 enzyme following LPS stimulation. Expression of the mRNA and protein was significantly reduced by pretreatment with AGR in a dose-dependent manner (Figure 2A,B). This study examined the effects of AGR on the secretion of inflammatory cytokines and showed that pretreatment with AGR strongly inhibited the secretion of both TNF-α and IL-6 and the expression of their respective mRNAs (Figure 1C–F). This effect was statistically significant and concentration-dependent at most treatment concentrations. Although the amount of IL-1β cytokine secretion in BV2 microglial cells was so low that it could not be detected by ELISA, mRNA expression, as analyzed by RT-qPCR, was strongly inhibited by AGR pretreatment as well as the expression of other cytokine genes (Figure 1G).

HO decomposes heme to produce biliverdin, CO, and ferrous ions. Both biliverdin and bilirubin have antioxidative effects. CO in the air is a toxic gas, but HO-1-induced CO has anti-inflammatory properties [20]. Therefore, the expression of HO-1 can represent antioxidative and anti-inflammatory action, which can affect the expression of iNOS induced by LPS in BV2 cells and may consequently inhibit NO secretion. Our results show that AGR extract effectively induces the production of HO-1 in BV2 microglia and has an inhibitory effect on the HO-1 induced production of NO and iNOS (Figure 2C,D). Nrf-2 is a pivotal regulator of several antioxidant enzymes, such as HO-1, and catalyzes the degradation of heme to antioxidants such as biliverdin, CO, and iron [21]. Nrf-2 liberated from cytoplasmic Kelch-like ECH-associated protein (Keap) 1 is transported into the nucleus and induces the production of HO-1, which plays an important role in inhibiting the inflammatory response. We also observed that the pattern of increase of Nrf-2 protein in the nucleus was similar to that of the production of HO-1 (Figure 2C), which appears to directly affect the neuroinflammation inhibitory activity of AGR.

NF-κB is a transcription factor that plays a crucial role in the expression of genes that regulate cellular activities such as inflammation and cell differentiation [22], is stimulated by TLR4 activation, and is separated from the inhibitor protein IκBα in the cytoplasm and translocated into the nucleus. NF-κB translocated into the nucleus binds to the promoter region of target genes and promotes the transcription of inflammatory substances such as inflammatory cytokines, adhesion molecules, and chemokines [23]. In this study, we also analyzed the expression of NF-κB pathway proteins, in order to investigate the mechanisms by which extract of AGR inhibits neuroinflammation. It appears that AGR has an inhibitory effect on the production of inflammatory substances, in view of the rapid decrease of cytoplasmic NF-κB p65 protein by LPS stimulation, partial regeneration by AGR pretreatment, and reduction of p65 protein migration into the nucleus due to the regulation of NF-κB activation (Figure 3A,B). Analysis of the expression of the inhibitory protein IκBα revealed that degradation and phosphorylation following LPS stimulation were inhibited by pretreatment with AGR, confirming that AGR has an inhibitory effect on NF-κB activation (Figure 3C).

MAPKs are serine/threonine kinases that transport extracellular signals into the nucleus, and their signal transduction leads to the synthesis of mediators that induce inflammatory responses. Phosphorylation of MAPK induces the activation of transcription factors including NF-κB and activator protein (AP)-1, involved in the formation of free radicals such as NO, and playing an important role in the activation of early stage inflammatory responses [24]. In BV2 microglia, phosphorylation of ERK, p38, and JNK MAPK by LPS was strongly suppressed by pretreatment with AGR, and showed high dependence on concentration (Figure 4). These results suggest that one of the major causes of the neuroinflammatory inhibitory activity of AGR is its regulation of MAPK activity.

We also identified the main components (nodakenin, decursin, demethylsuberosin, and chlorogenic acid) of the AGR extract through HPLC analysis (Figure 5). Previous studies of these components have shown that that nodakenin appears to have inhibitory effects on inflammation resulting from the regulation of TNF and NF-κB-related mechanisms in macrophages as observed by the inhibition of endotoxin shocks in mice [25]. There have also been reports that decursin inhibits the activation of MAPK and NF-κB induced by Aβ in PC12 cells [26]. Demethylsuberosin has also been reported to be effective in inhibiting the production of IL-6, NO and PGE_2_ in mast cells and macrophages [27,28]. These studies indicate that the antineuroinflammatory efficacy of AGR is closely related to the activity of its three constituents, nodakenin, decursin, and demethylsuberosin. In order to confirm the antineuroinflammatory activity of these components, we evaluated the effect of nodakenin, decursin, and demethylsuberosin on the secretion of NO and inflammatory cytokines on the microglia and confirmed that the three compounds showed antineuroinflammatory activity (Figure 6). In addition, the effects on the activation of NF-κB and MAPK were evaluated to explore the antineuroinflammatory mechanism of these components, and the results showed that the three components significantly inhibit the nuclear translocation of p65 protein, phosphorylation of IκBα, and phosphorylation of ERK, p38 MAPK (Figure 7). The antineuroinflammatory activities and structural features observed for the three main components showed that the original coumarin (demethylsuberosin; **3**) had stronger activity than furanocoumarin (nodakenin; **1**) and pyranocoumarin (decursin; **2**), which indicated that the coumarin skeleton plays an important role in anti-neuroinflammatory activity.

## 4. Materials and Methods

### 4.1. Materials and Reagents

Dulbecco’s Modified Eagle’s Medium (DMEM), fetal bovine serum (FBS), and antibiotics were purchased from HyClone (Logan, UT, USA). LPS from *Escherichia coli* O55:B5, bovine serum albumin (BSA), and DEX were purchased from Sigma–Aldrich (St. Louis, MO, USA). Consumables used for cell culture, including dishes, well plates, and tubes, were purchased from Sarstedt (Nümbrecht, Germany). CCK and ELISA antibody sets were obtained from Dojindo Molecular Technologies (Kumamoto, Japan) and eBioscience (San Diego, CA, USA), respectively. Primary antibodies and horseradish peroxidase (HRP)-conjugated secondary antibodies for Western blotting were acquired from Cell Signaling Technology (Boston, MA, USA). Polyvinylidene fluoride (PVDF) membrane for Western blotting was obtained from Millipore (Bedford, MA, USA). RNA extraction kits were purchased from iNtRON Biotechnology (Daejeon, Korea). DNA synthesis kits, oligonucleotide primers, and AccuPower^®^ 2X Greenstar qPCR Master Mix (ROX) were purchased from Bioneer (Daejeon, Korea). AGR ethanol extract was obtained as a freeze-dried powder from the Korea Plant Extract Bank (Ochang, Korea), dissolved in dimethyl sulfoxide (DMSO) and stored at −20 °C before use. Chemical standards for AGR (nodakenin, decursin, demethylsuberosin, and chlorogenic acid) were purchased from ChemFaces (Wuhan, China). Information on the various reagents and antibodies used in this study is shown in Table 2.

### 4.2. Microglial Cell Culture, Stimulation, and Treatment of Test Drugs

Mouse microglial BV2 cells were obtained from Prof. Kyoungho Suk at Kyungpook National University (Daegu, Korea). The BV2 cells were maintained in complete DMEM containing 1% antibiotic (100 U/mL penicillin and 100 μg/mL streptomycin) and 10% FBS. Cells were incubated in a humidified 5% CO_2_ atmosphere at 37 °C. All experiments were conducted 18 h after cell seeding in various culture plates. To stimulate the inflammatory responses of BV2 microglia, the culture medium was exchanged for fresh serum-free DMEM and 100 ng/mL of LPS was added in the presence or absence of AGR (10, 50, and 100 μg/mL) for the periods indicated.

### 4.3. Viability Assay for BV2 Microglia

To determine cell viability, BV2 microglia were plated in 96 well culture plates (5 × 10^4^ cells/well) and were incubated with different concentrations of AGR and LPS for 48 h. The CCK solution was then treated and incubated for an additional hour. Cell viability was calculated from optical density by analyzing the color changes in each well using an ELISA reader at a wavelength of 450 nm using a SpectraMax i3 (Molecular Devices, San Jose, CA, USA).

### 4.4. Analysis of NO Secretion

Griess assays were performed to analyze the effect of AGR on the secretion of NO. Microglia cells were seeded in 96-well cell culture plates under the same conditions used for the viability test, and preincubated for 18 h. Three concentrations of ARG were pretreated and after 1 h 100 ng/mL LPS was added to the culture to induce an intracellular inflammatory response. After another 24 h, 100 μL of Griess reagent was added to each well of the 96-well plate. After incubation at room temperature for 5 min, absorbance was measured at 570 nm using an ELISA reader, in order to evaluate the levels of expression or inhibition of NO.

### 4.5. ELISA for the Analysis of Inflammatory Cytokine Secretion

ELISA experiments were conducted to investigate the effect of AGR extract on the secretion of the inflammatory cytokines TNF-α and IL-6 induced by LPS in BV2 cells. Each well of a 24-well cell culture plate was seeded with 2.5 × 10^5^ cells. Preincubation and pretreatment of AGR and LPS treatments were performed under the same conditions as the previous Griess assay. After 6 h incubation, culture medium was retrieved, cell debris was removed by centrifugation, and the resulting medium used for ELISA analysis, performed according to the manufacturer’s standard protocol. The amount of each inflammatory cytokine was evaluated according to the intensity of its color after treatment with the TMB substrate, and the absorbance was read at a wavelength of 450 nm using an ELISA reader.

### 4.6. Preparation of Cytoplasmic and Nuclear Extracts for NF-κB and Nrf-2 Protein Detection

Cytoplasmic and nuclear extracts were separated according to the manufacturer’s protocol using NE-PER™ Nuclear and Cytoplasmic Extraction Reagents (Thermo Scientific, Rockford, IL, USA). Extracts were stored at −80 °C and processed using the same approach as the Western blotting of whole cell lysate.

### 4.7. Western Blotting for Protein Expression Measurement

To prepare samples for Western blotting, BV2 cells were seeded on a six-well plate at 1.5 × 10^6^ cells per well and cultured overnight. After replacement with fresh serum-free medium, the AGR extract was treated at different concentrations and cultured for 1 h, and an inflammatory response was induced by LPS. Cultured cells were harvested at predetermined times and washed twice with ice-cold phosphate-buffered saline (PBS). The supernatant was collected following centrifugation, and the cells were lysed in radioimmunoprecipitation assay (RIPA) lysis buffer (Millipore) and incubated on ice for 30 min. Following centrifugation at 15,000 rpm the supernatant was collected and normalized using Bradford’s method, followed by denaturation at 95 °C for 5 min. Protein samples were subjected to 10% sodium dodecyl sulfate–polyacrylamide gel electrophoresis (SDS-PAGE) and transferred to PVDF membranes using transfer buffer. The non-specific binding sites of the membranes were blocked with 3% BSA for 1 h at room temperature, and then incubated overnight with specific primary antibodies at 4 °C. The membranes were then washed with tris-buffered saline (TBS)-T containing 0.1% Tween 20 nonionic detergent, incubated with HRP-conjugated secondary antibodies for 1 h at room temperature, and then analyzed using a Chemidoc system (BIO-RAD), with an enhanced chemiluminescence (ECL) solution. Each protein band was normalized according to β-actin expression and quantified using the ImageJ software (https://imagej.nih.gov/ij/).

### 4.8. RNA Extraction and RT-qPCR

Total RNA was isolated using easy-BLUE^TM^ RNA (iNtRON Bio) extraction kits in accordance with the manufacturer’s protocol, and cDNA was synthesized from the purified RNA. A total of 1 μg of RNA was reverse transcribed into cDNA using AccuPower^®^ RT PreMix. The RT-qPCR oligonucleotide primers for mouse microglial cDNA are shown in Table 3 [29]. RT-qPCR reactions were performed in triplicate in 20 μL volumes with the following reagents: 0.3 μM final concentration of each primer with 1 μL each of forward and reverse primers, 10 μL of AccuPower^®^ 2× Greenstar qPCR master mix, 5 μL of template DNA, and 3 μL of RNase-free water. The following cycles were applied for the RT-qPCR of TNF-α, IL-6, IL-1β, iNOS, COX-2, HO-1, and β-actin: 40 cycles of 94 °C for 15 s and 60 °C for 1 min [29]. Amplification and analysis were performed using the QuantStudio 6 Flex Real-time PCR System (Thermo Scientific), and samples were compared using the relative CT method. Fold changes in gene expression were determined relative to a blank control after normalization to β-actin expression using the 2^−ΔΔCT^ method.

### 4.9. Sample Preparation for HPLC Analysis

All sample solutions were dissolved in methanol. Standard stock solutions of compounds were prepared at a concentration of 1 mg/mL and were then serially diluted with methanol to obtain a calibration curve of standard solutions at various concentration levels ranging from 12.5–200 μg/mL for nodakenin (1) and chlorogenic acid (4); 37.5–600 μg/mL for decursin (2); and 3.125–50 μg/mL for demethylsuberosin (3). AGR extract was prepared at a concentration of 5 mg/mL. All solutions for analysis were filtered through 0.45 μm RC membrane syringe filters (Sartorius, Germany).

### 4.10. Optimization of Chromatographic Conditions

HPLC analysis was performed using a Dionex UltiMate 3000 system (Dionex Corp., Sunnyvale, CA, USA) equipped with a binary pump, autosampler, column oven, and DAD. System control and data analysis were carried out using Dionex Chromelon software. Separation was carried out on a Luna C18 column (250 × 4.6 mm, 5 μm, Phenomenex, Torrance, CA, USA), with the column oven temperature kept at 30 °C, at a UV wavelength of 330 nm. The mobile phase consisted of water (solvent A) and acetonitrile (solvent B) with gradient elution of 0–5 min, 5–15% B; 5–12 min, 15–20% B; 12–30 min, 20–25% B; 30–40 min, 25–50% B; 40–60 min, 50% B; and 60–70 min, 50–80% B, at a flow rate of 1.0 mL/min. Before injection of the next sample, the column was re-equilibrated with the initial gradient of solvents for 10 min.

### 4.11. Validation of the Method

Specificity was assessed by comparing the chromatographic profile of the standard with that of the sample extract. Linearity was assessed by computing the correlation coefficient (*R*^2^) of the calibration curve for each compound at concentrations ranging from 3.125 to 600 μg/mL. Regression equations were calculated using the equation *y = ax ± b,* where *y* and *x* are the peak areas and concentrations of the sample, respectively.

### 4.12. Preparation of the Main Components of AGR

The main components of ARG were identified using HPLC analysis, as described above. Each compound was dissolved in 100% DMSO. The stock concentration of the three compounds nodakenin, decursin, and demethylsuberosin was 50 mM, and the intracellular concentrations applied were 1, 10, 50, and 100 μM, (DMSO concentrations of 0.2% or less). The effects of these compounds on the viability of BV2 cells was measured, and their inhibitory effects on the secretion of NO and inflammatory cytokines induced by LPS was confirmed.

### 4.13. Statistical Analysis

Each result is an averaged value obtained from at least three independent experiments, and is expressed as mean ± standard error of the mean (SEM). Statistical significance was determined using one-way analysis of variance followed by Dunnett’s test, after comparing each treated group and the LPS group. Statistical significance was defined as * *p* < 0.05, ** *p* < 0.001, *** *p* < 0.0001 (vs. LPS).

## 5. Conclusions

In this study using BV2 microglia, AGR showed strong inhibitory activity at relatively low concentrations (10–100 μg/mL) in neuroinflammatory responses to LPS stimulation. AGR extracts effectively reduced the production of the inflammatory mediator NO, cytokines, related synthetic enzymes, and mRNAs. Inhibition of neuroinflammation was also due to the blocking of NF-κB and MAPK activation and the induction of HO-1/Nrf-2 activation. The antineuroinflammatory effect of AGR appears to be closely related to the presence of three components, nodakenin, decursin, and demethylsuberosin. The physiological activity of AGR was one of the strongest among the plant extracts we investigated. Based on the above results, AGR extract has strong potential as a candidate treatment for various inflammatory diseases.

## Figures and Tables

**Table 1:** Regression data and contents of four compounds from AGR.

Analytes	Regression Equation	r^2^	Content (mg/g)
Nodakenin (**1**)	y = 0.4451x − 0.3459	0.9994	9.0498 ± 0.0241
Decursin (**2**)	y = 0.5195x − 2.1516	0.9998	77.4079 ± 0.1176
Demethylsuberosin (**3**)	y = 0.4192x − 0.1848	0.9996	1.5062 ± 0.00013
Chlorogenic acid (**4**)	y = 0.4863x − 2.0576	0.9997	5.3723 ± 0.00042

**Table 2:** Reagents, kits, and antibodies used in this study.

Classification	Product Name	Corporation	Product No.
Cell culture materials	DMEM medium	HyClone	SH30021.01
	FBS	HyClone	SH30084.03
	Antibiotics	HyClone	SV30010
Inducer	LPS	Sigma–Aldrich	L6529
Cell viability kit	CCK	Dojindo Molecular Technologies	CK04
ELISA antibody sets	TNF-α	eBioscience	88-7324-77
	IL-6	eBioscience	88-7064-77
RT-qPCR reagents	RNA extraction kit	iNtRON	17061
	DNA synthesis kit	Bioneer	K-2044-B
	qPCR master mix	Bioneer	K-6251
Western blot antibodies	iNOS	Cell signaling technology	2977
	COX-2	Cell signaling technology	4842
	β-actin	Cell signaling technology	8457
	HO-1	Cell signaling technology	82206
	Nrf-2	Cell signaling technology	12721
	TBP	Cell signaling technology	8515
	NF-κB p65	Cell signaling technology	3034
	P-IκBα	Cell signaling technology	2859
	IκBα	Cell signaling technology	4814
	P-ERK	Cell signaling technology	4377
	T-ERK	Cell signaling technology	9102
	P-p38	Cell signaling technology	9211
	T-p38	Cell signaling technology	9212
	P-JNK	Cell signaling technology	9251
	T-JNK	Cell signaling technology	9252
	Secondary anti-mouse	Cell signaling technology	7076
	Secondary anti-rabbit	Cell signaling technology	7074

**Table 3:** Primers used for RT-qPCR.

Target Gene	Primer Sequence
TNF-α	F: 5′-TTCTGTCTACTGAACTTCGGGGTGATCGGTCC-3′
	R: 5′-GTATGAGATAGCAAATCGGCTGACGGTGTGGG-3′
IL-6	F: 5′-TCCAGTTGCCTTCTTGGGAC-3′
	R: 5′-GTGTAATTAAGCCTCCGACTTG-3′
IL-1β	F: 5′-ATGGCAACTGTTCCTGAACTCAACT-3′
	R: 5′-CAGGACAGGTATAGATTCTTTCCTTT-3′
iNOS	F: 5′-GGCAGCCTGTGAGACCTTTG-3′
	R: 5′-GCATTGGAAGTGAAGCGTTTC-3′
COX-2	F: 5′-TGAGTACCGCAAACGCTTCTC-3′
	R: 5′-TGGACGAGGTTTTTCCACCAG-3′
HO-1	F: 5′-TGAAGGAGGCCACCAAGGAGG-3′
	R: 5′-AGAGGTCACCCAGGTAGCGGG-3′
β-actin	F: 5′-AGAGGGAAATCGTGCGTGAC-3′
	R: 5′-CAATAGTGATGACCTGGCCGT-3′

F—forward; R—reverse.
